# Hispanic Migrant Farm Workers' Attitudes Toward Mobile Phone-Based Telehealth for Management of Chronic Health Conditions

**DOI:** 10.2196/jmir.2500

**Published:** 2013-04-26

**Authors:** Matthew Price, Deborah Williamson, Romina McCandless, Martina Mueller, Mathew Gregoski, Brenda Brunner-Jackson, Eveline Treiber, Lydia Davidson, Frank Treiber

**Affiliations:** ^1^National Crime Victims CenterDepartment of Psychiatry and Behavioral SciencesMedical University of South CarolinaCharleston, SCUnited States; ^2^Technology Applications Center for Healthy LifestylesCollege of NursingMedical University of South CarolinaCharleston, SCUnited States; ^3^College of CharlestonCharleston, SCUnited States; ^4^College of MedicineMedical University of South CarolinaCharleston, SCUnited States

**Keywords:** mobile phone, hypertension, health care disparities, rural health

## Abstract

**Background:**

Mobile phone–based interventions present a means of providing high quality health care to hard-to-reach underserved populations. Migrant farm workers (MFWs) are among the most underserved populations in the United States due to a high prevalence of chronic diseases yet limited access to health care. However, it is unknown if MFWs have access to mobile phone devices used in mobile health (mHealth) interventions, or if they are willing to use such technologies.

**Objective:**

Determine rates of ownership of mobile devices and willingness to use mHealth strategies in MFWs.

**Methods:**

A demonstration of mHealth devices and a survey were individually administered to 80 Hispanic MFWs to evaluate use of mobile phones and mHealth devices and willingness to use such technologies.

**Results:**

Of the 80 participants, 81% (65/80) owned cell phones capable of sending and receiving health-related messages. Most participants (65/80, 81%) were receptive to using mHealth technology and felt it would be helpful in enhancing medication adherence, self-monitoring health conditions, and receiving quicker medication changes from their doctors (median scores ≥4 on 5-point Likert scales). Relations between age and attitudes toward using mHealth were not statistically significant.

**Conclusions:**

Hispanic MFWs have access to mobile phones and are willing to use mHealth devices. Future work is needed to comprehensively evaluate the degree to which these devices could be used.

## Introduction

Approximately 2 million migrant farm workers (MFWs) perform agricultural duties in the United States annually [1]. This group has been shown to be at increased risk for serious injury and chronic health conditions, including essential hypertension (EH) and type 2 diabetes [2-4]. Rates of uncontrolled EH are elevated in young adult MFW (33%), which is higher than the national average for Hispanics (23%) [5]. EH is particularly problematic because its symptoms often go unnoticed until the individual experiences a cerebrovascular or cardiac event [6]. As such, there is a clear need to improve the care of EH in this population given the high risk of complications.

A primary challenge in the treatment of chronic conditions in MFWs is identifying methods to offer timely and consistent access to care given the mobility of their occupation [2,7,8]. Repeated relocations typically spanning several states prevents this group from establishing long-term relationships with care networks, which has been associated with fragmented treatment, diminished care for chronic conditions, and poorer overall health outcomes [2]. Similarly, MFWs must navigate new health care systems in each location, including identifying care centers and pharmacies, and overcoming substantial transportation difficulties [9]. Most MFWs are non-English speaking (84%) [9], which can limit communication when interacting with primarily English-speaking providers. These issues prevent MFWs from accessing medications to manage chronic conditions, disrupt medication adherence schedules, and increase risk for more serious health outcomes. Innovative strategies to provide routine and seamless contact between patients and providers for MFWs in the treatment of EH and other chronic conditions are needed [10]. Migrant community health centers established through the Federally Qualified Health Center (FQHC) system have helped improve care, but these centers are often underutilized, suggesting that alternative care models are needed [11].

Mobile health (mHealth) technology is a viable option to facilitate timely communication between providers and patients with the goal of providing consistent care for MFWs. Such technologies can deliver automated summary reports of health conditions to health care providers and patients in real time (eg, blood pressure and glucose), deliver interventions targeting motivation for behavior change, and promote adherence to medication regimens. Recent findings indicate that mHealth programs are capable of enhancing patient control of chronic diseases, such as EH and type 2 diabetes [12,13]. Such interventions capitalize on the ability to communicate with patients without interfering with their routines. For MFWs, such tools could prove invaluable toward adherence to recommended treatment. Additionally, such approaches could sustain partnerships with the FQHC providers across their migratory work patterns.

A necessary first step in devising a novel care model is to determine the extent that the new population is willing to adopt the strategy [14]. Prior work with other populations has suggested that most individuals are receptive to the use of mobile phone-administered treatments for elevated blood pressure (BP) [6], heart failure alert [15], asthma [16], stroke recovery [17], multiple sclerosis [18], depression [19], and physical therapy [20]. Findings indicated that patients were enthusiastic to use such services (yet expressed the need for technical assistance if needed), were concerned with malfunctioning equipment, and were worried about the confidentiality of their information. Most of these studies have not involved low-income minority populations. George and colleagues [21] examined similar issues in underserved African Americans and Latinos and found that these groups had elevated concerns about confidentiality of information, quality of care received via a mobile device, and were less trusting of the medical community. Similarly, a recent study found that low socioeconomic status was associated with a reduced willingness to use an Internet-based monitoring system for EH [22]. These results indicate that disadvantaged groups, those who are most likely to benefit from mHealth because of reduced access, may be the most skeptical of this treatment strategy.

In MFWs, concerns regarding confidentiality of information, trust of medical care centers, and fear of poor quality of care are likely to be elevated. Given that many of these workers are undocumented, there are concerns about providing identifying and tracking information that could have adverse consequences. Many have concerns about sharing identifying information because of immigration status [2]. For example, such concerns have limited this group from seeking emergency care in critical situations. Additionally, MFWs have limited resources; therefore, they may not have regular access to mobile devices. Thus, it remains unknown whether migrant farmers have access to mobile devices or are willing to adopt mHealth technology for management of health care-related needs, despite the potential benefits.

The current study used a formal survey developed based upon previous measures administered to patients with varying chronic diseases and our findings from interviews with MFWs and their health care providers. We aimed to determine rates of mobile phone ownership including smartphones, utilization of various phone features, and awareness of mHealth technology within a sample of MFWs at 2 worksites in rural South Carolina. The participants were then given a brief overview of how various medical devices, such as weight scales, glucometers, and BP monitors, can be enabled to send data to a smartphone and then to a secure computer via transmission across cell towers. The demonstration of a prototype mHealth system for BP control was provided and acceptability and willingness to utilize such technology was assessed. The demonstration involved a brief explanation of what information would be transmitted to their doctor (BP, blood glucose, weight) and how to use the BP monitor. Based upon the rapidly expanding ubiquity of cell phone usage across ethnic groups, it was hypothesized that MFWs would have a high rate of ownership of mobile devices (>75%). Additionally, based on the outcomes of several other studies that demonstrated a positive attitude toward mHealth technology [6,15-17,20], it was hypothesized that MFWs with EH would be more willing to use the technology than those without a diagnosis. Lastly, it was hypothesized that those who use a wider array of features on their mobile device, such as sending text messages or downloading applications, would have more positive perceptions about mHealth.

## Methods

### Participants

The participants in this study were 80 MFWs stationed at 1 of 2 agricultural complexes in Charleston County, South Carolina, during the 2011 and 2012 spring harvesting seasons. Participants were predominately male (70%) with a mean age of 29.76 years (SD 9.82). All participants were Hispanic and spoke fluent Spanish.

### Measures

A questionnaire was designed to evaluate demographic information, EH status, and self-reported medication adherence. A series of 9 questions to assess attitudes toward mobile phone remote monitoring for chronic disease management was adapted from prior studies [6,15,23]. These items assessed willingness to use a mHealth service: (1) for EH and diabetes care in general, (2) if they were taught how to use the devices, and (3) if technical support was available. Additional questions assessed beliefs about the effectiveness of mHealth practices for EH and diabetes and concerns about confidentiality. Ratings were made for each question on a 5-point Likert scale with higher scores indicating increased willingness to use such technology. Internal consistency for the measure was excellent with alpha =.92. The tenth item queried their a priori awareness of health-related remote monitoring technology. The questionnaire was administered in an interview format in Spanish. Another native speaker was available to assist any participants who needed assistance.

### Procedure

The MFWs were approached in 8 groups of 6 to 12 participants in the residential location of their worksite over a 4-month period. No participants declined to participate. Participants were given a brief description and demonstration of a BP device with Bluetooth wireless technology (AND model 9025 BT) and a Motorola Droid X smartphone with an installed software application for BP signal reception and transfer to secure server. The survey was approved by the Institutional Review Board of the Medical University of South Carolina.

## Results

### Clinical Characteristics

Seventeen (21%) farmers had EH based upon a previous BP evaluation by an on-site physician, but only 7 (41%) of these 17 patients reported having received prescriptions for this condition. Those classified as EH had higher systolic/diastolic BP (systolic mean 140.6, SD 20.2; diastolic mean 86.5, SD 13.9) than non-EH participants (systolic mean 116.1, SD 13.5; diastolic mean 73.7, SD 10.8). Self-reported medication adherence using the Morisky scale revealed that among those with EH, only 5 of 17 (29%) were completely adherent within the past week (eg, did not miss a dose).

### Mobile Phone Utilization

Cell phone ownership among this sample was substantial with 81% (65/80) having access to 1 or more mobile phones. Over one-third (31/80, 39%) owned a smartphone capable of using mobile phone applications. A random subsample of 10 farmers who reported owning a smartphone were asked to show their phones so they could be verified as Internet-capable smartphones. All were verified as being smartphones with Internet connectivity. Text messaging was the most commonly used mobile phone feature of MFWs (62/80, 78%). Participants reported using their phones for email (28/80, 35%), accessing the Internet (36/80, 45%), and downloading applications (38/80, 48%). Few participants (12/80, 15%) had prior knowledge of the use of mobile phones in health care.

### Attitudes and Willingness Toward mHealth Technology

As shown in Table 1, most participants (65/80, 81%) reported they would likely or definitely use mHealth services if available. There was an increase in willingness if free technical support was offered (75/80, 94%). Most endorsed mHealth services as being helpful in maintaining timely linkages with their doctors (68/80, 84%) and reported having minimal or no doubts about the security of their health information on mHealth services (61/80, 76%). Security about health information was defined as the transfer of BP values, medication adherence, and other personal health information not being viewed by anyone other their health care provider team and themselves.

A series of multiple regressions were used to identify predictors and barriers toward positive perceptions of using mHealth services (Table 2). Effect sizes for each model were moderate with the multivariate coefficients of determination (*R* ^*2*^) ranging from 0.12 to 0.28. There were no differences in willingness to use mHealth across those with EH and those without EH. There were no significant predictors of willingness to use mHealth without assistance or willingness to use mHealth with a tutorial. Accessing the Internet via a phone was associated with increased willingness to use mHealth with continued technical support (beta=0.39, *P*=.02). Those who had prior knowledge of mHealth were less willing to continue to use the service with continued technical support (beta=–0.27, *P*=.02), were more likely to believe that mHealth can improve patient-provider communication (beta=0.38, *P*=.03), and were less concerned about confidentiality (beta=0.39, *P*=.02). The negative association between age and beliefs that mHealth can improve patient-provider communication did not achieve statistical significance (beta=–0.27, *P*=.05). Finally, women were less concerned about confidentially through mHealth (beta=0.34, *P*=.006).

**Table 1:** Responses to self-report survey assessing attitudes toward mHealth technology in migrant farm workers (N=80).

Questions assessing attitudes		Proportion of responses, n (%)
**Prior awareness of mHealth technology**		
	Yes	10 (13)
	No	70 (87)
**Willingness to use mHealth to manage essential hypertension (EH) and diabetes**		
	Definitely would not use it	4 (5)
	Not likely to use	3 (4)
	Neither likely nor unlikely	8 (10)
	Likely would use	37 (46)
	Definitely would use it	28 (35)
**Willingness to use a mHealth with initial tutorial on the application**		
	Definitely would not use it	2 (3)
	Not likely to use	3 (4)
	Neither likely nor unlikely	4 (6)
	Likely would use	34 (41)
	Definitely would use it	37 (46)
**Willingness to use mHealth with continued technical support**		
	Definitely would not use it	2 (3)
	Not likely to use	0 (0)
	Neither likely nor unlikely	3 (4)
	Likely would use	34 (43)
	Definitely would use it	41 (51)
**Confident mHealth can improve communication with provider about EH and diabetes**		
	No it cannot	2 (3)
	Yes, but have many doubts	2 (3)
	Not sure	8 (10)
	Yes but have some doubts	10 (13)
	Yes without a doubt	58 (71)
**Confident privacy protected when using mHealth system**		
	No trust	2 (3)
	Many doubts	5 (7)
	Neither trust nor distrust	10 (13)
	Few doubts	16 (20)
	Complete trust	45 (56)

**Table 2:** Predictors of attitudes toward mHealth technologies among migrant farm workers.

Variable	Willingness to use mHealth		Willingness to use mHealth with initial tutorial on the app		Willingness to use mHealth with continued technical support		Belief that mHealth can improve communication with provider		Concerns about confidentiality	
	β	*P*	β	*P*	β	*P*	β	*P*	β	*P*
Age	–0.02	.87	0.14	.29	0.05	.67	–0.27	.05	0.05	.70
Male gender	0.06	.62	0.11	.37	–0.02	.89	–0.01	.99	0.34	.006
Diagnosis of EH	<0.01	.99	0.06	.62	–0.03	.79	–0.12	.37	–0.03	.82
Own cell phone	0.24	.09	0.15	.29	0.22	.08	0.22	.12	0.13	.33
Own smartphone	–0.17	.26	–0.25	.10	–0.12	.41	–0.13	.42	–0.03	.84
Sends text messages	0.21	.13	0.32	.06	0.148	.23	–0.15	.28	–0.14	.28

## Discussion

The current study demonstrated that MFWs have high access to mobile devices. This suggests that the mHealth infrastructure, via short message service (SMS) text messages, phone calls, or emails, exists within this population. Over one-third (39%) had an Internet-capable smartphone which is consistent with the rising national average, as well as estimates of smartphone ownership among Hispanics from a current nationally representative sample (49%) [24]. This proportion is expected to steadily increase as such devices become more affordable and available on a growing number of flexible (month-to-month) plans. Current estimates project that virtually all cell phones in the United States will be smartphones with Internet access capability and Bluetooth-enabled within 1.5 to 2 years [25].

Most participants indicated they would use mHealth services (particularly if free technical support was available), believed it would be helpful for managing chronic diseases, such as hypertension and/or diabetes, and expressed few concerns about the security of their medical data across the Internet. Participants who indicated they had no prior knowledge of mHealth were more receptive to being involved in mHealth if they received an initial personal tutorial to help facilitate use of a mHealth service. This highlights a potential barrier: reluctance to engage in use of such technology due to lack of exposure and unfamiliarity with mHealth programs. Designers of mHealth applications would benefit from engaging patients in the development process to help ensure instructions are easily understood. Indeed, recent work has highlighted that problems caused by user error and technological errors are likely to increase frustration and prevent continued use of such approaches [16]. Incorporation of a patient-centered approach will further increase the likelihood of a user-friendly solution that will educate and encourage novice users to engage in the intervention [26-30]. However, additional work is needed to determine if such tutorials, which may be perceived as frustrating to those with higher levels of software proficiency, are a barrier to those with knowledge of such treatments.

There were no differences in any of the willingness measures across those with EH and those without EH. The perceived lack of need for continued care among those with such a condition may further highlight the need to develop mHealth programs that are user-friendly and minimally burdensome. Those with EH may not perceive the management of their condition as particularly relevant to their daily functioning [6]. Thus, mHealth systems cannot rely on participant motivation to ensure the use of such technology.

These findings suggest that mHealth is a promising method of providing health care to the difficult-to-reach group of MFWs. The findings from this study helped facilitate development of a recently activated 3-month proof-of-concept mHealth medical regimen enhancement trial among uncontrolled EH patients [31]. Electronic medication trays provide reminder signals and smartphone messages remind patients to measure their BP with the Bluetooth-enabled monitor used in the demonstration. Patients receive personalized motivational and reinforcement messages based upon adherence levels to the mHealth program. All who have received the mHealth program thus far have showed increases in medication intake (≥95% across the 3 months), reduced resting systolic BPs (from 163.8 to ≤120.9 mmHg at each monthly clinic evaluation and at 3-month follow-up), and reduced 24-hour systolic BP (from 151.3 to 122.7 mmHg at the completion of 3 months). These promising findings require completion of the proof-of-concept trial and subsequent refinement of the mHealth system based upon patient and provider feedback. This suggests that such a system should be evaluated with MFWs.

Although informative, the current findings must be interpreted cautiously. The sample size was relatively small compared with prior work [6,16]; it is unclear if these findings will apply across MFW communities and to other health care issues common to Hispanic MFWs [2]. Additional epidemiological work is needed to more fully gauge ownership of mobile phones in MFWs and attitudes toward the use of such approaches in medical care with empirically validated measures. Similarly, only attitudes were evaluated, and the extent these mHealth-driven interventions will truly be used is unknown, particularly within a framework of multisite, interlinked health centers, such as FQHCs. Finally, related work in this area has highlighted the need to identify provider attitudes to use such services [15]. Such work should focus on the willingness of providers to offer care to patients who they may be unable to contact face-to-face in the event of an emergency and the frequency with which contact should occur.

There are numerous barriers in managing chronic conditions among MFWs that mHealth has the potential to overcome. Remote access would allow MFWs and their primary providers to maintain contact and improve adherence to health maintenance behaviors [32]. The MFWs’ primary community health center provider teams, typically FQHCs, would be able to help navigate the MFWs as they migrate across geographical regions to gain access to other FQHCs and pharmacies that provide discounted or free medications. The consistent connection between patients and providers through the mHealth system would also likely enhance provider competence and cultural awareness for working with this group. A model of seamless interstate health care for MFWs as they traverse the eastern seaboard seems plausible, especially through collaboration with rural FQHCs. If future large-scale efficacy and effectiveness trials reveal similar results to the pilot work, the mHealth program for EH control will be ready for large-scale dissemination among MFWs. Such programs would vastly improve the health care of this highly vulnerable and underserved group. The data from the current study will support future work to develop culturally sensitive efficacious mHealth programs for this underserved population.

## Abbreviations

BP: blood pressure
EH: essential hypertension
FQHC: Federally Qualified Health Center
MFW: migrant farm workers
SMS: short message service
